# EPLIN: a fundamental actin regulator in cancer metastasis?

**DOI:** 10.1007/s10555-015-9595-8

**Published:** 2015-09-09

**Authors:** Ross J. Collins, Wen G. Jiang, Rachel Hargest, Malcolm D. Mason, Andrew J. Sanders

**Affiliations:** Cardiff China Medical Research Collaborative (CCMRC), Cardiff University School of Medicine, Henry Wellcome Building, Heath Park, Cardiff, CF14 4XN UK; Department of Clinical Oncology, Cardiff University School of Medicine, Cardiff, UK

**Keywords:** EPLIN, Cancer, Metastasis, Actin

## Abstract

Treatment of malignant disease is of paramount importance in modern medicine. In 2012, it was estimated that 162,000 people died from cancer in the UK which illustrates a fundamental problem. Traditional treatments for cancer have various drawbacks, and this creates a considerable need for specific, molecular targets to overcome cancer spread. Epithelial protein lost in neoplasm (EPLIN) is an actin-associated molecule which has been implicated in the development and progression of various cancers including breast, prostate, oesophageal and lung where EPLIN expression is frequently lost as the cancer progresses. EPLIN is important in the regulation of actin dynamics and has multiple associations at epithelial cells junctions. Thus, EPLIN loss in cancer may have significant effects on cancer cell migration and invasion, increasing metastatic potential. Overexpression of EPLIN has proved to be an effective tool for manipulating cancerous traits such as reducing cell growth and cell motility and rendering cells less invasive illustrating the therapeutic potential of EPLIN. Here, we review the current state of knowledge of EPLIN, highlighting EPLIN involvement in regulating cytoskeletal dynamics, signalling pathways and implications in cancer and metastasis.

## Introduction

The incidence of cancer is slowly rising and has become a global burden. A fundamental reason why cancer is such a problem is because of its ability to spread, invade surrounding tissue and potentially form secondary cancers at distinct sites around the body by metastasis. Cancer hallmarks include uncontrolled cell growth and evasion of cell death, and this ultimately can lead to tumour formation. According to the World Health Organisation (WHO), 8.2 million people died from cancer in 2012 worldwide [1]. In the UK alone, mortality rates reached 162,000 annual deaths [2]. This illustrates a considerable need for better treatment, diagnosis and management of the disease. Epithelial protein lost in neoplasm (EPLIN) is a molecule involved in regulation of the actin cytoskeleton and has been implicated in the development and progression of various cancer types, displaying frequent downregulation or loss in cancer, creating a potential for prognostic targeting and as a tumour suppressor. This current review discusses EPLIN’s role in actin dynamics and in the pathophysiology of cancer development and progression.

## Epithelial protein lost in neoplasm

**Fig. 1:**
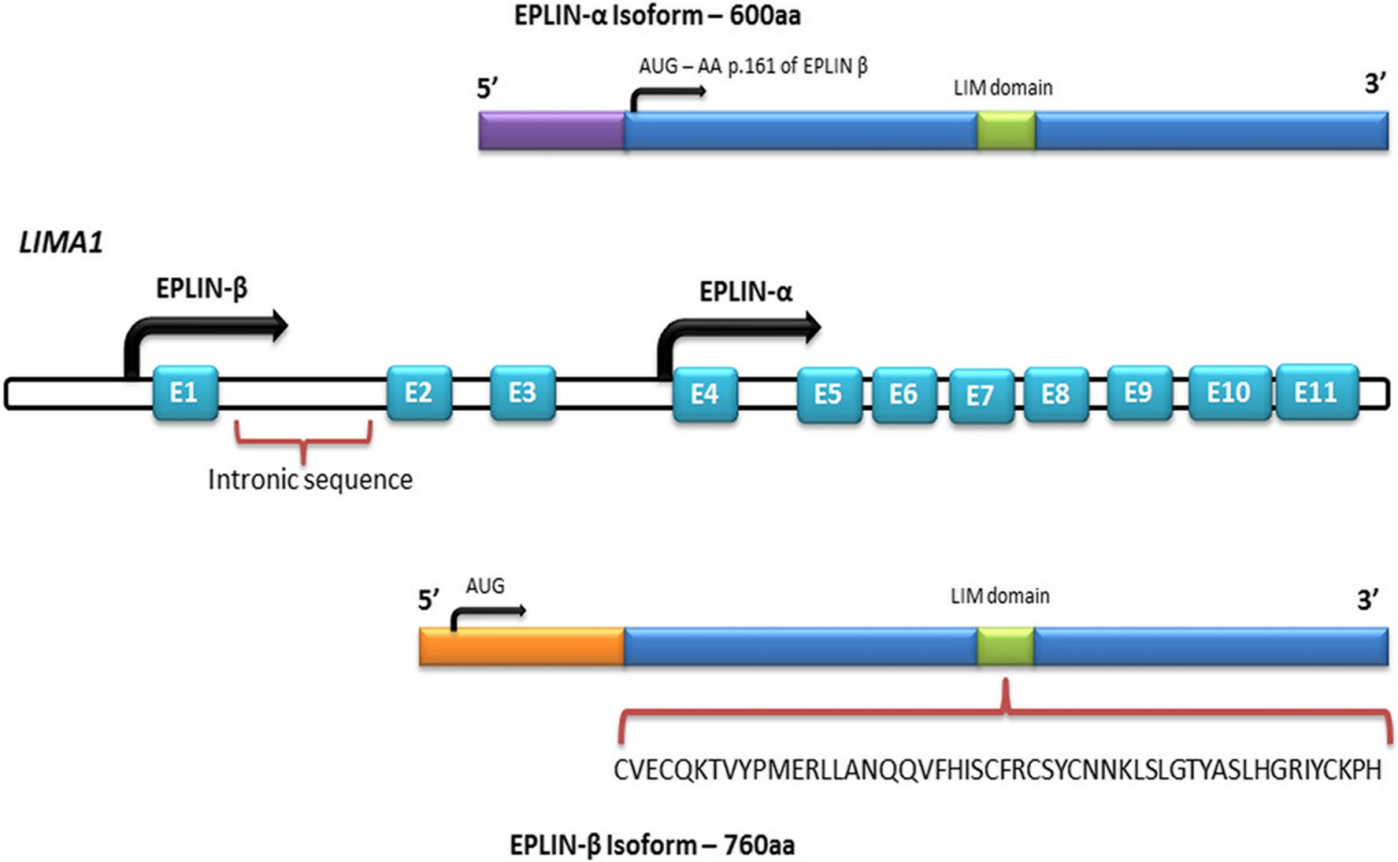
Schematic diagram of the *LIMA1* genomic structure and EPLIN structural isoforms. The *LIMA1* gene consists of 11 exons and ten introns. EPLINα differs from EPLINβ at the amino terminus where an additional 160 amino acids are present in EPLINβ. Shown below EPLINβ is the 52-amino acid centrally located LIM domain common to both EPLIN isoforms. Adapted from [4]

**Fig. 2:**
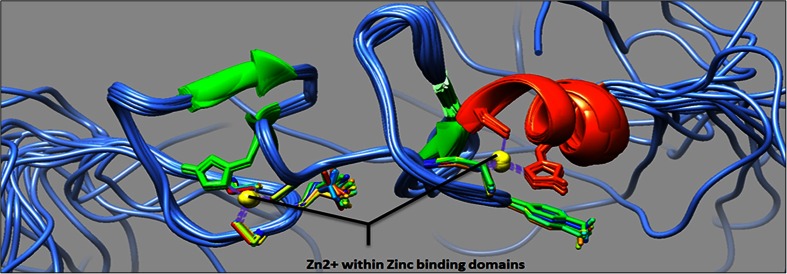
Protein structure of EPLIN LIM domain (PDB ID=2D8Y). Protein structure of the EPLIN centrally located LIM domain. Zinc-binding domains depicted. This domain may aid self-dimerisation. Image generated using UCSF Chimera software

**Fig. 3:**
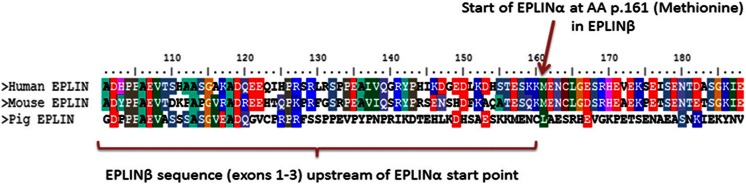
ClustalW protein alignment of human, mouse and pig EPLINβ. Areas of amino acids that are conserved across species are *highlighted*. The region shown is amino side of the EPLINβ protein, where EPLINα originates at amino acid (AA) p. 161. ClustalW generated using BioEdit Biological Sequence Alignment software

EPLIN is a cytoskeletal, actin-binding protein encoded by the *LIMA1* gene. EPLIN was initially identified in oral cancers for its differential expression between normal oral epithelial cells and human papilloma virus (HPV)-immortalised oral epithelial cells [3]. EPLIN exists as two distinct isoforms, a 600 amino acid EPLINα isoform and a larger 759 amino acid EPLINβ isoform, generated from an alternative pre-mRNA splicing event (see Fig. 1) [4]. The EPLINα isoform has been implicated in the progression of various cancers, and this was initially recognised in oral cancer, breast, prostate and xenograft tumours where EPLIN expression was either downregulated or completely abolished [4]. The amino acid sequence of EPLIN is characterised by a single centrally located LIM domain which supposedly aids structural self-dimerisation and contains subdomains for zinc binding (see Fig. 2) [4, 5]. LIM-domain-containing proteins are frequently present in molecules responsible for cytoskeletal organisation, such as the focal adhesion phosphoprotein, paxillin [6]. EPLIN is important in the regulation of actin dynamics and aids actin filament bundle assembly, and the amino terminal of the EPLIN protein structure is essential for this localisation to the actin cytoskeleton [7]. The EPLIN genomic structure consists of 11 exons and ten introns, with exons 1–3 only present in EPLINβ and EPLINα utilising exons 4–11 of *LIMA1* for transcription [5]. The EPLIN gene has two separate promoter regions; the EPLINβ promoter is near the start of the gene in exon 1, whilst EPLINα initiates ∼50 kb downstream near the end of intron 3, prior to exon 4 and at amino acid position 161 in the EPLINβ protein (see Fig. 1) [5]. Sequence analysis has revealed that EPLIN is conserved across species with EPLINα and EPLINβ isoforms present in mouse, displaying 77 and 75 % identity similarity for human EPLINα and EPLINβ, respectively (see Fig. 3) [8]. A role for EPLIN has also been suggested in muscle development in pigs, where EPLIN displayed a temporal expression pattern with only the EPLINα isoform present in developing skeletal muscle [9]. Since the discovery of EPLIN, our lab has shown that aberrant EPLIN expression is associated with the progression of various cancer types including breast, oesophageal, pulmonary and prostate cancer. EPLINα levels decrease as the cancer progresses and becomes more advanced, giving EPLINα potential to provide prognostic value, and overexpression analysis suggests that EPLINα is a putative tumour suppressor [10–14]. The described loss of EPLIN in cancer has functional implications on the actin cytoskeleton and may contribute to enhanced metastatic potential of cancer cells.

### The epithelial protein lost in neoplasm interactome: regulation in actin dynamics

**Fig. 4:**
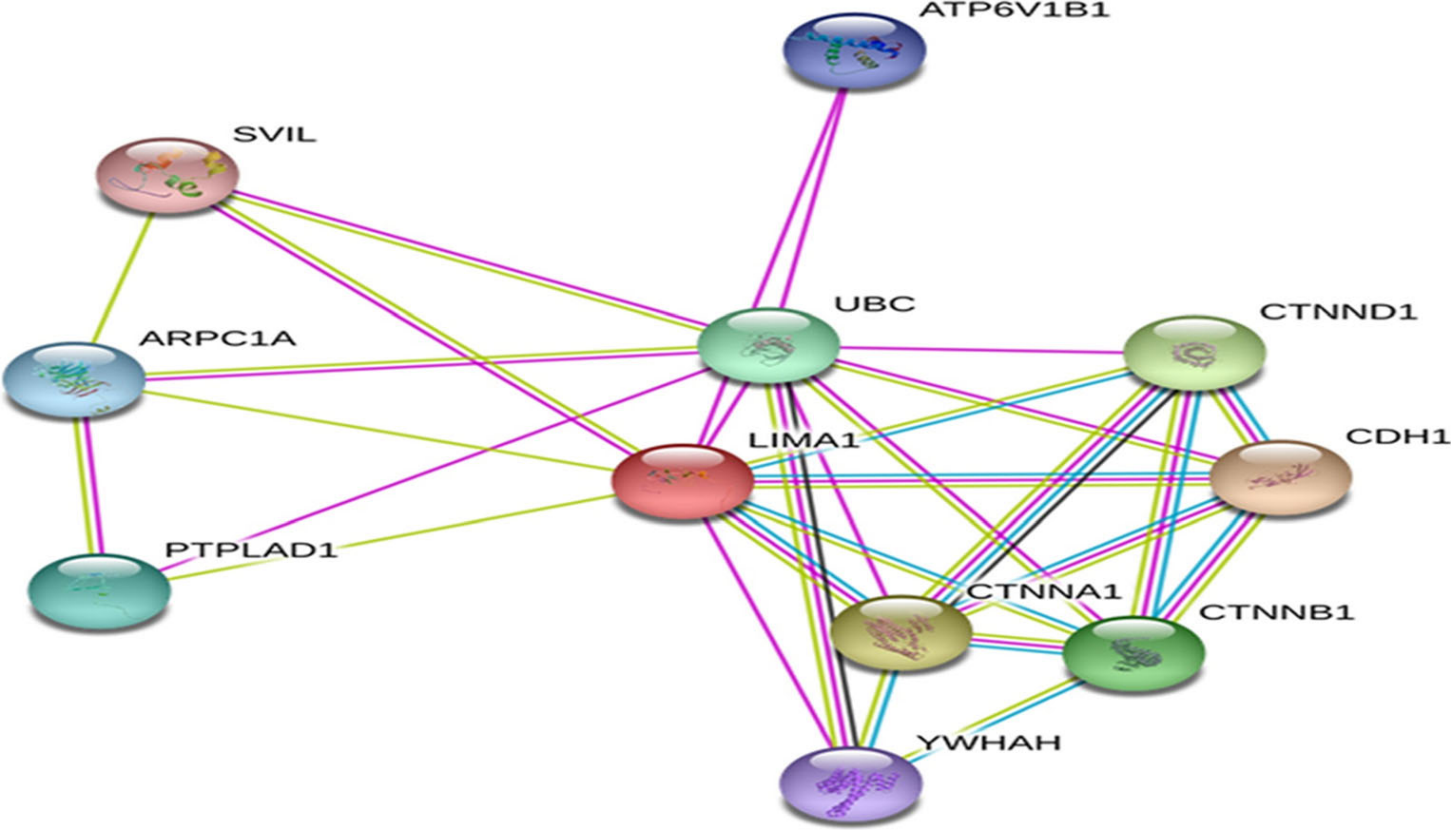
EPLIN predicted functional partners. EPLIN (LIMA1) has various associations including cadherin and catenin molecules which contribute to cytoskeleton regulation. *LIMA1* LIM domain and actin binding 1; *CDH1* cadherin 1; *CTNNA1* catenin-α1; *CDH1* cadherin 1; C*TNND1* catenin-δ1; *CTNNB1* catenin-β1; *UBC* ubiquitin C; *PTPLAD1* protein tyrosine phosphatase-like A domain-containing 1; *ARPC1A* actin-related protein 2/3 complex, subunit 1A; *ATP6V1B1* ATPase, H+ transporting, lysosomal 56–58 kDa, V1 subunit B1; *YWHAH* tyrosine 3-monooxygenase/tryptophan 5-monooxygenase activation protein; *SVIL* supervillin. Image generated and extracted from online STRING database

EPLIN has a number of functional partners (see Fig. 4/Table 1), and the globular protein actin is central to the function of EPLIN. EPLIN has two functional actin-binding sites which flank the central LIM domain, and it is this binding capacity that engenders actin cross linking and actin filament bundle assembly [15]. A fibrillar pattern is displayed by both isoforms, and expression of EPLINα enhances the size and number of actin filament stress fibres and can also inhibit membrane ruffling via the signalling GTPase, Rac1 [15]. EPLIN therefore directly interacts with actin which suggests a possible role for EPLIN in cell migration, adhesion and cell morphology. Actin is an abundant, multifunctional protein responsible for cell migration in eukaryotic cells. Actin is part of the cytoskeletal network which consists of microtubules, microfilaments and intermediate filaments which are vital for cellular functions. Actin exists as monomers (G-actin) and filamentous polymers (F-actin) and is important for physiological functions including cell locomotion, cytokinesis, maintenance of cell shape and muscle contraction [24]. Transcription of the *LIMA1* gene is suggested to be primarily controlled by monomeric G actin, with the actin–MAL–SRF signalling pathway regulating EPLIN production [25]. Maul *et al*. [15] illustrated that EPLINα has three significant features: EPLINα has at least one binding site for actin and can cross link and bundle actin filaments, EPLINα stabilises actin filaments *in vitro*, and EPLINα inhibits branching nucleation of actin filaments by the Arp 2/3 complex [15]. Therefore, this suggests that EPLIN may orchestrate actin filament dynamics by stabilising actin cytoskeletal networks [15]. Additionally, EPLIN has been shown to form part of an actin-remodelling complex composing of EPLIN, β-actin, γ-actin and gelsolin which co-localises at the plasma membrane to the tumour suppressor, phosphatase and tensin homolog (PTEN) [26]. PTEN is a well-established tumour suppressor molecule, so this asks the question of whether the interaction of PTEN and the actin-remodelling complex is itself an element suppressing the development of neoplastic tissue and whether any interruption of these complexes may promote cancer progression. Based on these findings, EPLIN accommodates actin to accomplish various actin-related cellular processes including cell motility and migration and cell junctional adhesion [17]. There is increasing evidence to suggest that EPLIN regulates actin structures in cooperation with the signal transduction adaptor protein, paxillin. When EPLIN is overexpressed, paxillin exhibits an increased staining pattern for both human endothelial cells line (HECV) and PC-3 cells [12, 13]. This co-localisation pattern is also observed in cultured human mesangial cells at focal adhesion sites, and co-immunoprecipitation results confirm an association between the two molecules [16]. EPLIN and paxillin may form a complex and potentially stabilise focal adhesions to co-ordinate actin dynamics in a complimentary manner. Given EPLIN’s role in actin dynamics, it is strongly implicated in cellular processes including cell migration and invasion, and thus, downregulation or loss of EPLIN expression in cancer may likely affect the metastatic potential of cancer cells. Actin and EPLIN are located in epithelial cells at the adherens junction (AJ) and contribute to functional cellular adhesion between adjacent cells.

**Table 1:** EPLIN-associated molecules

EPLIN association	Biological significance and reference	References
Actin	Actin is an abundant protein important for cell migration. EPLIN has two actin-binding domains that flank the EPLIN LIM domain. Pull-down assays revealed that EPLIN binds actin monomers, and this results in actin cross linking and actin filament bundle assembly.	[15]
Paxillin	May form a complex with EPLIN to co-ordinate actin dynamics. IHC of PCa tissue *vs* normal reveals that EPLIN overexpression influences paxillin expression and localisation. Co-localisation, co-precipitation and an *in situ* proximal ligation assay revealed direct association between the two molecules in cultured human mesangial cells.	[12, 13, 16]
α-Catenin	Immunoprecipitation and GST pull-down assays reveal that EPLIN interacts with α-catenin, forming a cadherin–β-catenin–α-catenin–EPLIN complex.	[17]
Supervillin	*In vivo* co-localisation studies and *in vitro* GST pull-down assays reveal that EPLIN interacts with the peripheral membrane protein, supervillain.	[18]
PINCH-1	Pull-down assays reveal that endogenous EPLIN co-immunoprecipitates with endogenous PINCH-1 in keratinocytes.	[19]
ERK	ERK phosphorylates EPLIN and decreases EPLIN affinity to F-actin promoting cell migration. Inhibition of ERK abolishes EPLIN expression and reduces tumour-suppressive ability of EPLIN.	[10, 13, 20]
DNp73	In melanoma cells, both EPLIN isoforms are inhibited by DNp73, and this drives a more invasive phenotype.	[21]
SATB2	EPLIN is differentially regulated by the DNA-binding protein, SATB2. SATB2 regulates the actin cytoskeleton via EPLIN association. When SATB2 is knocked out, osteosarcoma cells show reduced migration and are less invasive, and this is mediated by EPLIN.	[22]
Cav-1	EPLIN regulates the lipid raft tumour-suppressive protein, Cav-1. Co-immunoprecipitation and mass spectroscopy analysis revealed that EPLIN and Cav-1 bind to each other in normal and RasV12 cells.	[23]

### The adherens junction

**Fig. 5:**
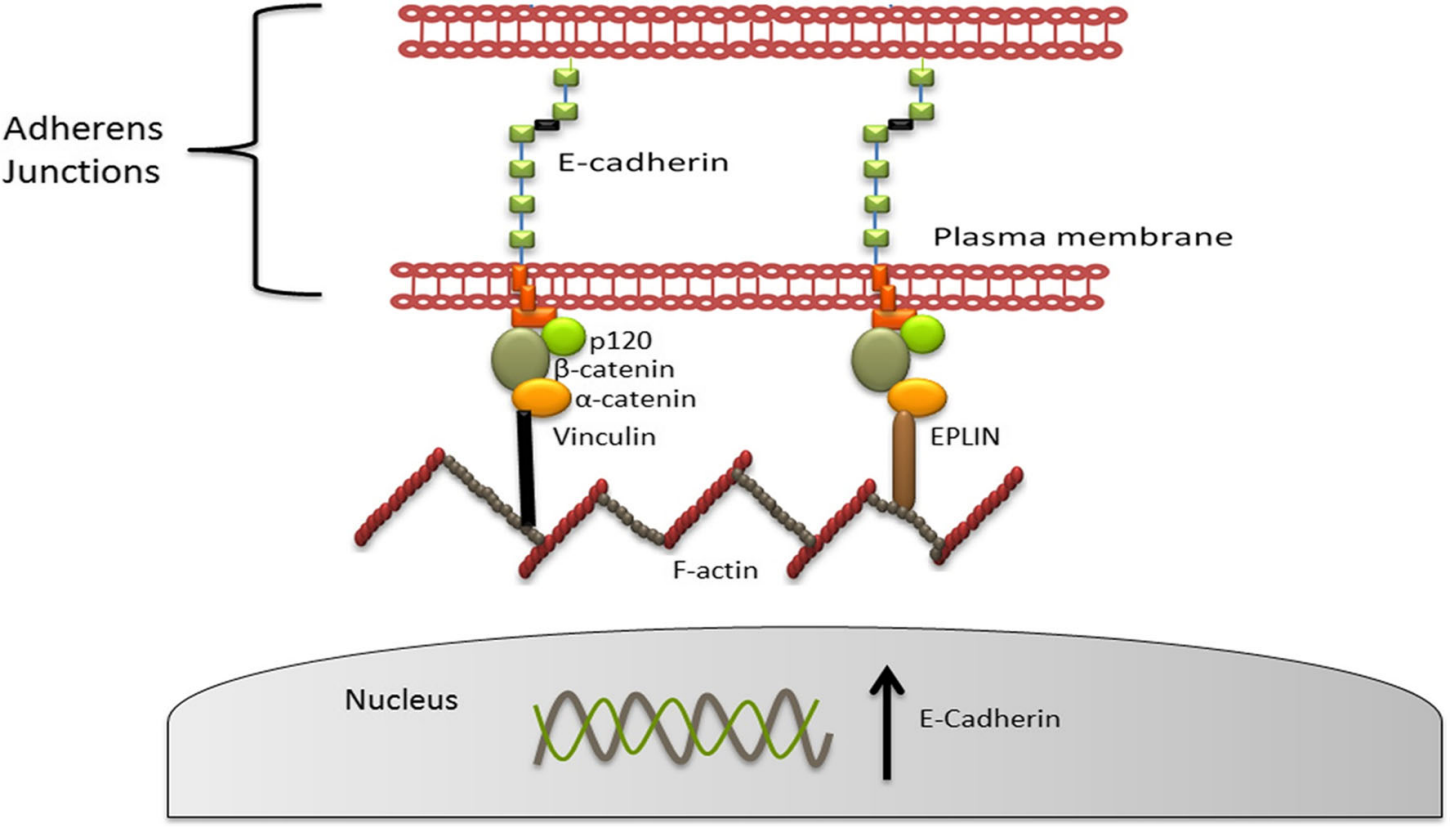
Schematic representation of adherens junctions. The AJ between epithelial cells consists of various protein complexes to orchestrate actin cytoskeletal dynamics. The cadherin–catenin complex is associated with filamentous actin via EPLIN and/or vinculin which binds α-catenin in the cytoplasm. Adapted from [27]

The AJ is a type of anchoring junction found predominantly in epithelial cells, also referred to as the zonula adherens, which functionally link the actin cytoskeleton together in cells via linker molecules. EPLIN is an actin-binding protein, which functions to bundle actin filaments; therefore, EPLIN presence is required at AJ along with filamentous actin. AJ contains various protein complexes along with EPLIN, and these include cadherins, catenins and p120 catenins (see Fig. 5) [28]. Within the AJ, cadherins and catenins associate together to form the cadherin–catenin complex and EPLIN provides a direct physical link for this complex to the actin cytoskeleton [17]. The cadherin–catenin complex is composed of E-cadherin, β-catenin and α-catenin, with E-cadherin positioned between adjacent cells and the catenins positioned in the cytoplasmic space of each cell [29]. E-cadherin binds directly to β-catenin which sequentially binds α-catenin, generating the cadherin–catenin complex (see Fig. 5) [30]. α-Catenin is a crucial player at the AJ and was initially recognised as responsible for providing the bridge between the cadherin–catenin complex and actin [28, 31]. This principle, however, has come under scrutiny, as direct *in vitro* binding between α-catenin and actin has never been detected [32, 33]. Cavey and co-workers [34] realised that α-catenin is not essential for E-cadherin stability, as complexes of α-catenin and E-cadherin were detected in RNAi α-catenin embryos [31]. Therefore, it is apparent that cadherin–actin interaction is regulated not only by α-catenin but by a number of actin-binding proteins that are associated with α-catenin, including EPLIN [30, 35]. Abe and Takeichi [17] demonstrated, by immunoprecipitation and GST pull-down assays, that both EPLIN isoforms directly interact with the α-catenin VH3 plus C-terminal region to generate a cadherin–catenin–EPLIN–actin complex at cell junctions [17]. When EPLIN is depleted, the bridge to F-actin was unable to form due to loss of organisation of the apical actin belt, with punctate accumulation of E-cadherin at cell junctional points [17]. This illustrates the importance of EPLIN in producing functional epithelial junctions. Additional molecules involved in regulating the AJ include the membrane cytoskeletal protein, vinculin, and the actin filament-binding protein, afadin [36, 37]. Recent work from Taguchi *et al*. [35] illustrated that EPLIN and vinculin may collaborate together in AJ formation via binding α-catenin either together or individually and cooperatively aid junctional adhesion [35]. The cooperation of EPLIN and vinculin in cellular adhesion is also evident in endothelial cells. At the endothelial AJ, the endothelial E-cadherin homolog, VE-cadherin, interacts with β- and γ-catenins, which sequentially bind α-catenin and EPLIN in an analogous fashion to epithelial cells [38]. This allows the recruitment of vinculin and ultimately promotes strengthening of inter-endothelial junctions [38]. The authors propose a role for EPLIN in tension dissemination at the endothelial AJ in a mechanosensory mechanism [38]. The machinery from actomyosin exerts tension through EPLIN, which causes α-catenin to adopt a more accessible conformation, revealing a vinculin-binding site and allowing vinculin recruitment and actin association at endothelial cell–cell AJ [39]. This mechanotransduction mechanism consisting of EPLIN and α-catenin suggests that the endothelial AJ is regulated in a spatial and temporal fashion [39]. Finally, EPLIN also appears to be important for attachment to F-actin in endothelial cells; when EPLIN expression is downregulated, the organisation of F-actin is considerably disrupted, leading to multiple holes in the actin cytoskeleton [38]. Altogether, this suggests that the AJs of epithelial and endothelial cells are orchestrated by various actin-binding, α-catenin-associated molecules and are dynamically regulated, with EPLIN having a critical role in cell adhesion, creating further implications of EPLIN loss in cancer.

### Epithelial protein lost in neoplasm—a key player in cell division?

Cell division is the splitting of one cell into two, where biological information is passed onto daughter cells. For this process to successfully occur, various proteins need to functionally regulate the division and these include Rho GTPases, cyclin-dependant kinases, integrins, cdc42, focal adhesion kinases, myosin and the globular protein actin [40]. With this in mind, an actin-binding protein like EPLIN may potentially have a regulatory role in cell division. This has been recently shown using HeLa cells where EPLIN depletion resulted in large numbers of multinucleated cells, signifying cytokinesis failure during cell division [41]. In successful mitotic division, actin and myosin II accumulate at the cleavage furrow during cytokinesis and EPLIN loss compromised each protein’s ability to efficiently do this [41]. EPLIN appears to be important for the accumulation of other mitotic regulatory proteins including the GTPases RhoA and cdc42, where EPLIN depletion resulted in either a significantly reduced concentration of RhoA or a misplaced location of cdc42 at the cleavage furrow [41]. EPLIN aids this successful cell division in conjunction with a number of regulatory proteins including supervillin and the oncogenic kinesin, KIF14, suggesting a complex network of regulatory proteins at the cleavage furrow [18]. Altogether, this suggests that EPLIN may have an integral role in cytokinesis and loss may lead to aneuploidy and genomic instability of daughter cells [41]. Therefore, EPLIN is crucial to co-ordinate actin and myosin dynamics throughout cell division and loss in cancer cells could have downstream effects on successful cytokinesis, increasing their tendency to form a cancer [41].

### Post-translational modification

**Fig. 6:**
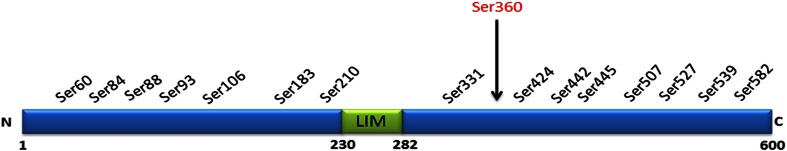
Predicted phosphorylation sites in EPLINα. The protein structure of human EPLIN has multiple putative phosphorylation sites at all regions of the protein, including various sites where serine kinases would likely act. Predicted threonine and tyrosine phosphorylation sites not shown. The phosphorylated residue suggested in [20] is indicated. Phosphorylation status predicted using NetPhos 2.0 software

Extracellular signal-regulated kinase (ERK) is a member of the mitogen-activated protein kinase (MAPK) family and is important in the regulation of actin organisation by phosphorylating various proteins including paxillin, focal adhesion kinase (FAK) and other protein kinases and nuclear transcription factors to co-ordinate cellular processes [42, 43]. ERK is implicated in cellular events including cell migration and may facilitate this by phosphorylation of actin-bundling proteins like EPLIN [20]. The protein structure of EPLIN has multiple putative phosphorylation sites (see Fig. 6), and it has been shown that ERK phosphorylates EPLIN at Ser360, Ser602 and Ser692 *in vitro* and *in vivo* [20]. Phosphorylation at the carboxy terminal of EPLIN decreases affinity to F-actin and thus provokes a reorganisation of the actin cytoskeleton, enhancing cell migration [20]. This implicates ERK in actin organisation and cell motility with EPLIN being a critical substrate for phosphorylation [20]. A recent study by Zhang *et al*. [44] illustrated that this ERK-mediated phosphorylation of EPLIN is itself regulated by epidermal growth factor (EGF) and revealed how targeting this signalling cascade can be manipulated to reduce epithelial–mesenchymal transition (EMT) and, thus, prostate cancer invasiveness [44]. ERK also plays a role in targeting EPLIN to focal adhesions and effects the interaction with paxillin; activation of the MEK–ERK pathway both reduced localisation of EPLIN to sites of focal adhesions and abolished paxillin interaction [16]. These data suggest that ERK is functionally related to EPLIN and provides a critical regulatory role for appropriate actin dynamics and may have implications in cancer progression.

## The role of epithelial protein lost in neoplasm in cancer

Cancer progression involves various cellular, morphological and molecular alterations which result in a transformed cellular phenotype, ultimately having the potential to invade surrounding tissue and disseminate throughout the body. Cancer treatment options remain largely unspecific and create various undesired side effects. Therefore, elucidating a molecular target for treating cancer, in addition to understanding the mechanism of cancer development, is crucial. EPLIN first received attention for its involvement in cancer in 1999 where EPLIN downregulation was described in various cancer cell lines [4]. Altogether, low levels of EPLIN transcript were found in 8/8 oral cancer cell lines, 5/6 breast cancer cell lines and 4/4 prostate cancer cell lines [4]. Using PC-3 and DU-145 prostate cancer cell lines, EPLIN expression was significantly reduced compared to primary prostate epithelial cells (PrEC), whereas the prostate specific antigen (PSA) positive LNCaP and LAPC4 prostate cancer cell lines failed to express EPLINα at all [4]. This notion of EPLIN loss is also seen in breast cancer where EPLIN expression in cell lines BT-20, SKBr-3, MCF-7, T-47D and MDA-MB-231 was either reduced or completely lost [4]. Lastly, the authors demonstrated EPLIN as a putative tumour suppressor molecule, where overexpression of EPLINα caused a reduction in cancer cell growth [4]. Interestingly, when EPLINα was depleted in breast cancer cell lines, EPLINβ either remained consistent or actually increased [4]. This illustrates the potential cancer protective effects that the EPLINα isoform may exert in various cancer cell systems. EPLIN overexpression has also proved effective in altering the growth phenotype and morphology in additional cell systems including anchorage-independent NIH3T3 transformed cells [7]. Using a soft agar assay and utilising the activated Cdc42 or the chimeric nuclear oncogene EWS/Fli-1 to transform NIH3T3 cells, EPLIN overexpression resulted in a ∼80 % decrease in colony formation for Cdc42 transformed cells, with a similar growth decrease in EWS/Fli-1 transformed cells [7]. Interestingly, EPLIN displayed heterogeneous staining throughout Ras cells rather than localisation to the actin cytoskeleton [7]. This implies that oncogenic transformation affects the EPLIN/actin architecture, and thus, the localisation of EPLIN to the actin cytoskeleton may be important to exert its suppressive ability [7].

### Prostate cancer

**Fig. 7:**
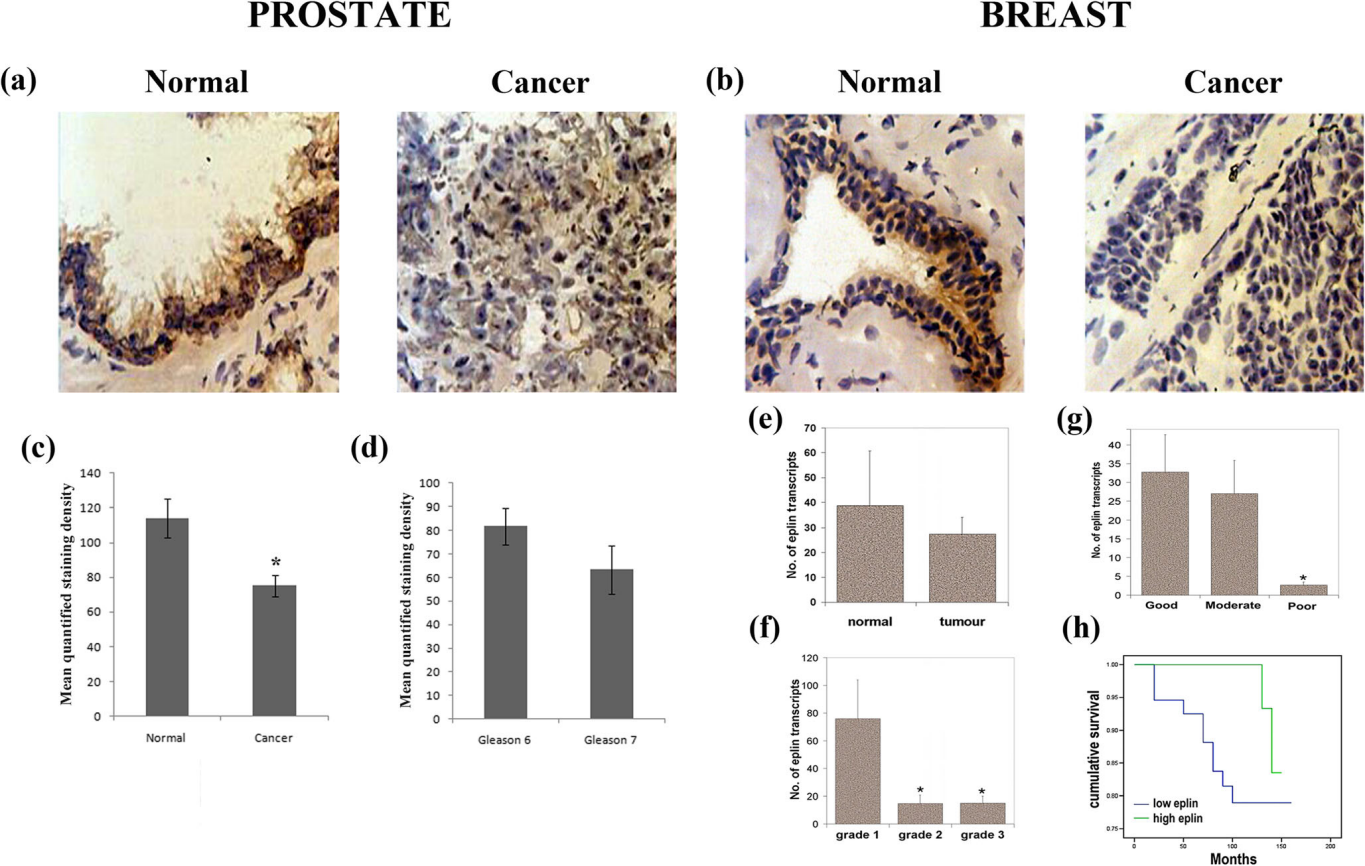
EPLIN profile in clinical prostate and breast cancer. Immunohistochemical staining (×20 objective magnification) of normal and cancerous **a** prostate and **b** breast clinical samples demonstrating EPLIN localisation and expressional differences. **c**, **d** Semi-quantitative analysis of EPLIN staining within prostate clinical cohort demonstrates that lower levels of EPLIN staining are associated with cancerous and higher-grade samples. **e** Within a clinical breast cancer cohort, lower transcript expression of EPLIN is seen in tumour samples compared to normal breast tissue and was associated with a higher grade (**f**), a poorer patient prognosis (**g**) and reduced overall survival rates (**h**). Figure modified from [10, 12]

There is increasing evidence to suggest that EPLIN is implicated in the development of prostate cancer and the process of EMT. EMT is a process where polarised epithelial cells are downregulated and subjected to biochemical and morphological changes. Epithelial cells can become transformed to a mesenchymal cell phenotype, losing their cell polarity and cell adhesion at cellular junctions [45]. The converted mesenchymal cell phenotype has a reorganised cytoskeleton and experiences alterations in cell signalling which engenders enhanced migratory and invasiveness capabilities and increased resistance to apoptosis [45, 46]. During these cellular changes, the actin molecular architecture is disrupted and protein complexes like the cadherin–catenin complex and epithelial markers are lost [47]. EPLIN is associated with the cadherin–catenin complex and contributes to functional cytoskeletal dynamics [17]. Zhang and co-workers [47] used biochemical and functional approaches to demonstrate that EPLIN is a negative regulator of EMT and invasiveness in prostate cancer (PCa) cells. EPLIN was significantly decreased in cells of more mesenchymal morphology (known as the androgen refractory cancer of the prostate (ARCaPM) cell lineage model), suggesting that EPLIN downregulation is directly implicated in EMT, along with the cadherin–catenin complex [47]. Depletion of EPLIN also provokes various other morphological changes including disassembly of AJ, increased migratory and invasive potential of cells *in vitro*, activation of β-catenin signalling, increased expression of vimentin, increased chemoresistance and decreased expression and nuclear translocation of E-cadherin [47]. Lastly, the authors used immunohistochemistry to show that EPLIN downregulation is correlated with cancer progression in multiple cancer models including lymph node metastasis in PCa, where EPLIN expression was significantly reduced [47]. Altogether, this illustrates that EPLIN may be involved in the regulation of EMT and PCa progression and loss of EPLIN can lead to diverse downstream cellular effects. EPLIN in PCa has also been recently evaluated by our laboratory using the classical PCa cell line, PC-3. By immunohistochemistry (IHC), EPLIN displayed a significant decrease in staining for tumour cells in comparison to normal (see Fig. 7a) [12]. This is accompanied by quantification of staining intensity within the cohort, where lower levels of EPLIN staining are associated with cancerous and higher-tumour-grade samples (see Fig. 7c, d) [12]. Overexpression analysis of EPLINα resulted in decreased growth rate in tumour cells *in vitro*, along with reduced invasiveness and cell adhesion to the extracellular matrix (ECM) [12]. Mice injected with PC-3 cells overexpressing EPLINα developed tumours at a markedly decreased rate in comparison to control [12]. Furthermore, cells overexpressing EPLINα displayed a greater staining pattern for the focal adhesion targeting protein, paxillin, implying that EPLIN may also be present at these plaques [4, 12]. Altogether, these results demonstrate the potential of EPLIN for monitoring PCa progression and how it can be manipulated to suppress PCa via impeding cancerous traits.

### Breast cancer

Our lab has recently evaluated EPLIN involvement in cancer progression in a number of model systems [10–14]. By comparing EPLINα IHC staining in normal *vs* tumour cells in breast cancer progression, EPLINα was found to be substantially weaker in tumour cells than in normal epithelial cells (see Fig. 7b) [10]. This correlated with lower EPLINα transcript in tumour samples compared to normal samples (see Fig. 7e) with lower EPLIN levels being associated with higher tumour grade, a poorer patient prognosis and reduced overall survival rates (see Fig. 7f–h) [10]. IHC analyses in breast cancer from additional research groups also show EPLIN loss as the tumour becomes more aggressive, specifically comparing EPLIN immunointensity of primary tumours *vs* tumours with lymph node metastases [47]. Lastly, *in vitro* and *in vivo* overexpression analysis of EPLIN highlighted significant reductions in cell growth and cell invasion using transfected breast cancer cell lines, and also, highly significant reductions in tumour size were observed in nude mice inoculated with EPLIN-α-transfected *vs* control MDA-MB-231 breast cancer cells [10].

### Further pathological implications

Clinical implications for EPLIN also include oesophageal and pulmonary cancer (Table 2). Q-PCR analysis displayed reduced expression of EPLINα in an oesophageal cancer cohort for both cancerous tissue and cancer cell models [11]. With regard to tumour histological grade, tumour–node–metastasis (TNM) status, nodal status and survival status, EPLINα transcript generally decreased with levels significantly lower in patients who ultimately died from the cancer, suggesting that EPLINα is implicated in oesophageal cancer progression and may give an indication for cancer prognosis [11]. Overexpression analysis of EPLINα in the KYSE150 cell line resulted in decreased growth and invasiveness compared to normal, suggesting that EPLINα has tumour-suppressive ability by regulating cellular aggressiveness in oesophageal cancer [11]. In a pulmonary cancer cohort also conducted by our lab, Q-PCR analysis showed a reduction of EPLINα expression in tumour *vs* normal samples, where EPLIN was also reduced in later TNM stages and cancers with lymph node involvement [14]. Using the SKMES-1 cell line, overexpression of EPLINα via transfection in the pEF6 expression vector inhibited cell growth and cell motility [14]. In addition to the apparent molecular loss of EPLIN in various cancers, EPLIN also appears to be reduced at the protein level for colorectal cancer and squamous cell carcinoma of the head and neck (SCCHN), where IHC analysis revealed that EPLIN is decreased in cancers with lymph node metastases *vs* primary tumours [47].

**Table 2:** Summary of EPLIN clinical implications

Clinical implication	Description	Reference
Prostate cancer	(1) Immunoblot analyses demonstrated that EPLIN expression in prostate cancer cell lines and xenograft tumours is reduced compared to prostate epithelial cells (PcEC). PC-3, DU-145, LNCaP, LAPC4, LAPC3 and LAPC9 all displayed loss of EPLIN protein. (2) IHC analyses of normal and cancerous clinical prostate sections revealed a greatly reduced staining pattern of EPLIN in tumour samples. Overexpression of EPLIN in PC-3 cells negatively impacted cell growth *in vitro* and *in vivo*, and were less invasive and had reduced adhesion to the ECM. (3) EPLIN is implicated in the process of EMT, and IHC analysis revealed that EPLIN loss is correlated with prostate cancer progression, with a significant reduction of EPLIN expression in tissues with lymph node metastases compared to primary tumours and normal prostate tissues.	(1) [4] (2) [12] (3) [10, 47]
Breast cancer	(1) Immunoblot analysis revealed reduced or abolished expression of EPLIN protein in tumourigenic breast cancer cell lines (BT-20, SK-Br-3, MCF-7, T-47D and MDA-MB-231) compared to mammary epithelial cells (MEC), immortalised mammary epithelial cells (IMEC) and a non-tumourigenic breast cancer cell line, HBL-100. (2) Analysis by Q-PCR revealed lower levels of EPLINα in tumour samples compared to normal. Higher-tumour-grade samples had lower EPLIN transcript. Patients with poorer prognosis and patients who died of the cancer had significantly lower levels of EPLIN transcript. Overexpression of EPLIN rendered cells less invasive, and had a reduced growth rate *in vitro* and *in vivo* and were less motile. (3) IHC analyses displayed a reduction in EPLIN staining in tissues of breast cancer lymph node metastases compared to primary breast tumours.	(1) [4] (2) [10] (3) [47]
Oesophageal cancer	Q-PCR analyses revealed lower levels of EPLINα transcript in tumour tissues compared to normal. Higher-tumour-grade samples had lower EPLIN transcript. Patients who died of the cancer had significantly lower levels of EPLIN transcript. Patients with local advanced T stage cancer (T2–T4) and patients with lymphatic metastasis had lower levels of EPLINα transcript. Overexpression analysis caused cells to be less invasive and to have a reduced growth rate *in vitro* and *in vivo*.	[11]
Pulmonary cancer	Q-PCR analyses revealed reduced levels of EPLINα transcript in tumour samples compared to normal. Tissues of a higher TNM stage and where there was nodal involvement also had lower EPLIN transcript. Overexpression analysis revealed a reduction of cell growth and motility in the SKMES-1 cell line.	[14]
Colorectal cancer	IHC analyses revealed that EPLIN is significantly reduced in lymph node metastatic tumours compared to primary tumours in colorectal cancer.	[47]
SCCHN	IHC analyses revealed a reduction of EPLIN staining of cancerous tissue with lymph node metastasis compared to primary tumours.	[47]
Oral cancer	Northern analyses determined that EPLIN expression in 8/8 oral cancer cell lines is reduced compared to control G3PDH.	[4]

Collectively, these studies suggest EPLIN may be a clinical indicator for cancer progression in addition to providing further evidence of a tumour-suppressive role for EPLINα in the regulation of cancer progression.

Finally, in addition to the implication of EPLIN in the spread and progression of cancer, a recent publication provides a link between EPLIN and renal diseases where patients with either membranoproliferative glomerulonephritis (MPGN) or IgA nephropathy had a decreased expression profile for EPLIN via IHC analysis [16]. This advocates the idea that EPLIN may be involved in the pathology of various disease states.

### Angiogenesis

Angiogenesis is the formation of new blood vessels from pre-existing vessels and is essential for wound healing and normal growth and development. The angiogenic process is frequently utilised by cancer cells, by a means of metastasis, to reach secondary sites around the body and develop secondary tumours. Angiogenesis is therefore a critical factor when targeting cancer therapies. EPLINα demonstrates a suppressive role in angiogenesis, where overexpression analysis in the HECV endothelial cell line resulted in a reduced capacity to generate tubular structures in a Matrigel tubule formation assay when compared to vector controls [13]. This regulatory effect was also apparent *in vivo* where mice injected with HECV cells overexpressing EPLINα in conjunction with cancer cells developed tumours significantly slower than controls [13]. Forced expression also appears to exert an effect on cell matrix adhesion and migration capabilities in this cell line where cells overexpressing EPLINα both migrated at a significantly slower rate and were significantly less able to adhere to the Matrigel basement membrane [13]. This suppressive role in angiogenesis illustrates that EPLINα has potentially various regulatory mechanisms for reducing cancer metastasis and could be an effective target for cancer therapy.

## Conclusions and outlook

**Fig. 8:**
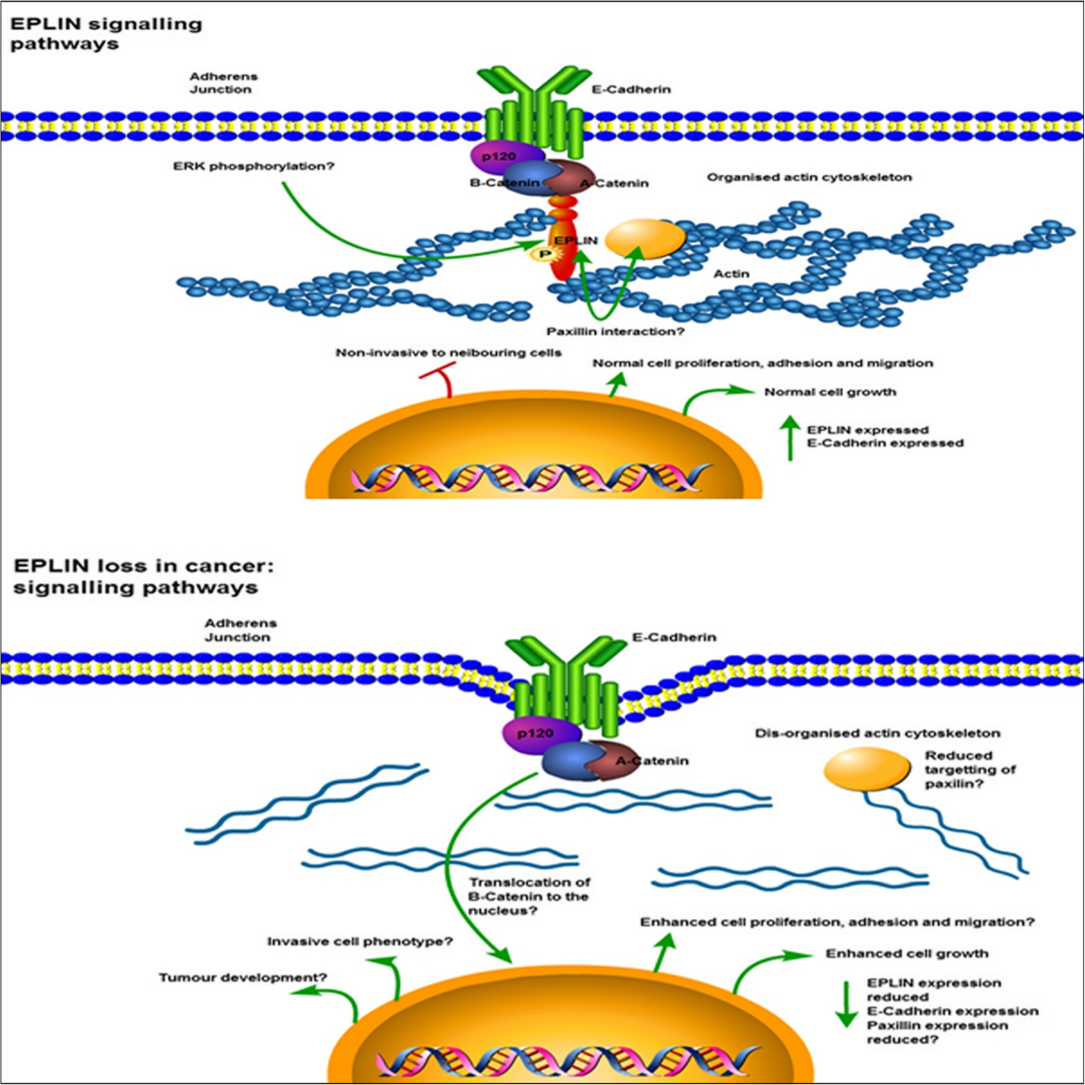
Proposed EPLIN signalling pathways and implications for loss in cancer. When cancer is not present, EPLIN associates with the actin cytoskeleton linking the cadherin–catenin complex to F-actin via interaction with α-catenin. The signal transduction protein, paxillin, interacts with EPLIN in the cytoplasm, and this complex likely stabilises actin dynamics. ERK phosphorylates EPLIN regulating cell motility and migration. When cancer is present and EPLIN is lost, the actin cytoskeleton becomes less organised and this induces membrane ruffling. Paxillin targeting is likely lost reducing focal adhesion between the cadherin–catenin complex and actin. These molecular, cellular and morphological consequences may result in increased metastatic potential including enhanced cell migration and motility. Signalling pathways summarised from [12, 16, 20, 47]. Image generated using Pathway Builder 2.0 software

The interaction of EPLIN and actin has provided an excellent model for investigating multiple aspects of cancer progression over the last decade. The discovery of EPLIN led to a subtle paradigm shift in structural view and organisation of cytoskeletal dynamics, with the acknowledgment that various actin-related molecules contribute to multiple dynamic processes underlying cellular migration and invasion. There is an established link between EPLIN and cancer progression with frequent downregulation of EPLIN in more aggressive cell lines, reduced staining in cancerous tissue samples and reduced growth potentials when there is forced expression of the EPLINα isoform *in vitro* and *in vivo*. EPLIN is functionally linked to molecules like actin and paxillin and has been implicated in a number of potential pathways to enhance metastatic potential (outlined in Fig. 8). However, the precise mechanistic action of EPLIN and, subsequently, how EPLIN loss contributes to the development of cancer remain elusive. Mechanistic investigations will therefore be crucial to elucidate the full importance of EPLIN in cancer pathophysiology.
